# Joint Effect of Multiple Common SNPs Predicts Melanoma Susceptibility 

**DOI:** 10.1371/journal.pone.0085642

**Published:** 2013-12-31

**Authors:** Shenying Fang, Jiali Han, Mingfeng Zhang, Li-e Wang, Qingyi Wei, Christopher I. Amos, Jeffrey E. Lee

**Affiliations:** 1 Department of Surgical Oncology, The University of Texas MD Anderson Cancer Center, Houston, Texas, United States of America; 2 Department of Dermatology, Brigham and Women’s Hospital, Harvard Medical School, Boston, Massachusetts, United States of America; 3 Channing Division of Network Medicine, Department of Medicine, Brigham and Women’s Hospital, Harvard Medical School, Boston, Massachusetts, United States of America; 4 Department of Epidemiology, Harvard School of Public Health, Boston, Massachusetts, United States of America; 5 Department of Epidemiology, The University of Texas MD Anderson Cancer Center, Houston, Texas, United States of America; 6 Geisel College of Medicine, Community and Family Medicine, Dartmouth College, Lebanon, New Hampshire, United States of America; Ohio State University Medical Center, United States of America

## Abstract

Single genetic variants discovered so far have been only weakly associated with melanoma. This study aims to use multiple single nucleotide polymorphisms (SNPs) jointly to obtain a larger genetic effect and to improve the predictive value of a conventional phenotypic model. We analyzed 11 SNPs that were associated with melanoma risk in previous studies and were genotyped in MD Anderson Cancer Center (MDACC) and Harvard Medical School investigations. Participants with ≥15 risk alleles were 5-fold more likely to have melanoma compared to those carrying ≤6. Compared to a model using the most significant single variant rs12913832, the increase in predictive value for the model using a polygenic risk score (PRS) comprised of 11 SNPs was 0.07(95% CI, 0.05-0.07). The overall predictive value of the PRS together with conventional phenotypic factors in the MDACC population was 0.69 (95% CI, 0.64-0.69). PRS significantly improved the risk prediction and reclassification in melanoma as compared with the conventional model. Our study suggests that a polygenic profile can improve the predictive value of an individual gene polymorphism and may be able to significantly improve the predictive value beyond conventional phenotypic melanoma risk factors.

## Introduction

The incidence rate of melanoma has substantially increased among all white populations during the past several decades[1]. In 2013 in the United States, about 76,690 new cases of melanoma are expected to be diagnosed, and 9,480 patients will die of this disease[2]. Melanoma is highly curable if discovered and treated early. Melanoma patient outcome varies significantly by stage; the overall 5-year survival rates for patients with localized melanoma or regional spread are approximately 98% and 62%, respectively, while for patients with distant metastatic disease, the 5-year survival rate is only about 15%[3,4]. Recent data suggests that systematic skin cancer screening can potentially reduce melanoma-associated mortality; broad population-based screening programs based on clinical evaluations of largely low risk individuals, however, are likely to be prohibitively expensive [5]. Therefore, an effective risk assessment tool is needed to help primary care providers identify individuals at high risk for melanoma. Risk factors for melanoma include a family history of the disease, fair pigmentation, multiple nevi, immunosuppression, intermittent exposure to ultraviolet radiation, and genetic determinants [6]. Familial aggregation of melanoma indicates that high-penetrance variants in melanoma susceptibility genes underlie predisposition to melanoma in some patients. Identified high-penetrance variants for melanoma risk include cyclin-dependent kinase inhibitor 2A (*CDKN2A*), cyclin-dependent kinase 4 (CDK4), an alternate reading frame (ARF) of *CDKN2A*, and a locus on 1p22 [7,8]. However, these rare variants appear to be responsible for only a small proportion of melanoma susceptibility. Candidate gene association studies and genome-wide association studies (GWASs) have identified several additional much more common but lower penetrance gene variants that are associated with both pigmentation or nevi and melanoma risk. Melanocortin 1 receptor (*MC1R*) is associated with red hair, pale skin, freckling, and sun sensitivity [9,10] and contributes to melanoma risk through both pigmentary and nonpigmentary effects [9]. The oculocutaneous albinism type II (*OCA2*) gene region on chromosome 15 demonstrates strong associations with eye and hair color [11,12] and an association with melanoma risk[13,14]. Additional pigmentation-related genes associated with melanoma risk are tyrosinase (*TYR*); tyrosinase-related protein 1 (*TYRP1*); solute carrier family 45, member 2 (*SLC45A2*); and solute carrier family 24, member 4 (*SLC24A4*) [12,13,15]. A region between *MTAP* and *CDKN2A* is associated with both the number of cutaneous nevi and melanoma risk [6,16]. A haplotype of the agouti signaling protein (*ASIP*) locus that is associated with nevus count also significantly influences melanoma risk[17–19]. Furthermore, some variants associated with melanoma risk have no demonstrated association with nevi or pigmentation phenotype[20]. These relatively common individual genetic variants identified from previous candidate gene association studies or GWASs generally have only weak effects on melanoma risk (odds ratios [ORs] < 1.3)[6,17,18,20,21]. We hypothesized that joint analysis of multiple single-nucleotide polymorphisms (SNPs) could detect a larger effect than those of the individual SNPs, and potentially improve the predictive power of models based on conventional phenotypic risk factors. To test our hypothesis, we sought to validate the association of melanoma with 11 previously identified SNPs in data sets from The University of Texas MD Anderson Cancer Center melanoma case-control study (MDACC), and the Harvard Medical School Nurse Health Study (NHS) and Health Professionals Follow-Up Study (HPFS), and to assess the predictive performance of these combined variants, including relative to conventional phenotype risk factors. 

## Methods

### Design of the MD Anderson Cancer Center Study

The original discovery study was a hospital-based, case-control investigation of cutaneous melanoma, for which non-Hispanic white patients and controls were recruited at The University of Texas MD Anderson Cancer Center between March 1998 and August 2008. After genotyping samples from 3,156 participants, we determined that 1,804 melanoma patients and 1,026 cancer-free controls (friends or acquaintances of patients reporting to other clinics) had adequate SNP data for analysis. The study protocols were described previously [21]. All individuals gave informed consent under The University of Texas MD Anderson Cancer

Center Institutional Review Board System (UTMDACC IRB System) review board-approved protocol. Participants provided their written informed consent to participate in this study. 

Patients with all stages of cutaneous melanoma evaluated in the Melanoma and Skin Cancer Center at MD Anderson were eligible for inclusion. Data on sex, age, race, skin color, hair color, eye color and tanning ability, and clinical prognostic factors were collected from patient records. Pigmentation information was collected from 931 patients and 1026 controls via questionnaire. 

### Genotyping and Data Quality Control

The current study used high-density genotype data obtained from 3,156 DNA samples of our melanoma patient and control populations. The samples were genotyped by using the Illumina HumanQmnil-Quad_v1-0_B array and details of genotyping information and data quality control were described in our previous study[21]. Finally, 818,237 genotyped SNPs remained for the primary analysis. Imputation of ungenotyped SNPs was performed using MACH[22] applied to genotype data from all subjects. In total, we had 2,649,586 imputed or directly genotyped SNPs eligible for an association study. Among them, we extracted 11 SNPs that demonstrated association with melanoma risk in previous studies for the current analysis. These 11 SNPs were selected using the method described in the Statistical Analysis section below. For the 11 SNPs included in our final data analysis, four (rs13016963, rs1335510, rs10830253, rs4911442) were imputed and seven were directly genotyped. The most significant SNP rs12913832 was directly genotyped. 

### Population from Harvard Cohort Studies

Both NHS and HPFS and their initial GWAS have been described in previous studies [15,21]. Finally, we included 494 CM cases and 5628 controls.

Based on the genotyped SNPs and haplotype information in the NCBI Build 35 of phase II Hapmap CEU data, we imputed genotypes for 2.5 million SNPs using the program MACH. In the validation study, we used the same 11 previously identified melanoma risk SNPs as described in the discovery dataset to observe their joint effect on melanoma risk.

Information on natural hair color at age 20 years was collected in both the NHS and HPFS prospective questionnaires, and information on natural eye color was collected in the HPFS only. Tanning ability was collected in NHS only. Hair color was divided into four categories (1= blonde ; 2=red; 3= brown ; and 4=black), while eye color was divided into three categories (1=blue/grey; 2=hazel/green; 3=brown/dark). Tanning ability was grouped into four categories (1=practically none; 2=light tan; 3=average tan; and 4=deep tan). 

### Statistical Analysis

We used PubMed (http://www.ncbi.nlm.nih.gov/pubmed?myncbishare=mdacclib&holding =mdacclib_fft) to search the literature using the key words “melanoma and susceptibility and GWAS” or “melanoma and genetic variants” to identify candidate gene association studies or GWASs published prior to December 2012; the studies found through this search identified a total of 38 SNPs associated with melanoma risk. These SNPs and their individual-SNP association results in the MDACC data set are summarized in Table S1 in File S1. For gene regions in which multiple SNPs were associated with melanoma susceptibility, we selected the most significant SNP (lowest *P*-value) within that gene region as representative to minimize the effect of linkage disequilibrium among the SNPs we intended to combine to create a risk score. In total we identified 15 independent candidate SNPs; 11 of these candidate SNPs were available for analysis in all 3 data sets. We counted the number of risk alleles for the selected SNPs within each individual, weighted by risk coefficients (Table S2 in File S1), and we determined their joint effect on melanoma susceptibility as described below.

We first performed logistic regression modeling to measure the additive effect of each individual SNP on melanoma susceptibility, without adjustment for any covariates. We then combined multiple SNPs by summing the number of risk alleles (0, 1, or 2) of each SNP, weighted by the effect sizes reported in previous studies, to obtain a weighted polygenic risk score (PRS). The analysis was first run in the discovery data set (MDACC), with adjustments for sex, age, skin color (1-3=light, 4-6=medium, 7-10=dark), hair color (1 = blonde, 2 = red, 3 = brown and 4 = black), eye color (1 = blue/gray, 2 = hazel/green and 3 = brown/black) and tanning ability (ordinal trait 1 to 5), and the analysis was then replicated in the NHS data set with adjustments for age, tanning ability, skin color, and melanoma family history; and in the HPFS data set with adjustments for age, eye color, hair color, and melanoma family history. A random effects model was used to pool the 3 data sets in a meta-analysis with inverse variance weight. The Cochran Q test and I^2^ index were used to evaluate heterogeneity across the 3 studies[23]. Corrections for multiple testing were made using Bonferroni adjustments. 

To further assess the predictive value of PRS in the model, we compared the discrimination of the following models: ①the most significant SNP rs12913832, ② PRS, ③sex and age, ④sex, age, and pigmentation, and ⑤ sex, age, pigmentation, and PRS. We evaluated the ability of the risk prediction models to distinguish those who had disease from those who did not using the area under the receiver operating characteristic curve (AUC), for which higher values indicate better discrimination. Increment in AUC in a model with conventional risk factors and the PRS, compared with conventional risk factors alone, was tested through the logistic regression procedure using SAS software (SAS Institute, Cary, NC)[24]. Study participants were divided into 3 categories (<20%, 20%-50%, and ≥ 50%) based on predicted probabilities with or without PRS. We then calculated the percentage of individuals who were reclassified into higher- or lower-risk groups using model ⑤ rather than model ④ to evaluate the usefulness of adding PRS to the standard phenotypic model. To evaluate the significance of novel biomarkers, we further calculated the integrated discrimination improvement(IDI) and the net reclassification improvement(NRI) as proposed by Pencina et al[25]. The IDI estimates the new model’s improvement in average sensitivity without sacrificing average specificity. The NRI estimates the change of reclassification of subjects based on their predicted probabilities of events using the new model with the option of imposing meaningful risk categories.

All statistical analyses were performed using SAS Enterprise Guide 4.3. All *P* values were 2 sided, and *P* values less than 0.05 were considered statistically significant.

## Results

### Baseline Characteristics of Melanoma Patients and Controls

A total of 8,950 participants—2,829 from MDACC (one individual with missing age information excluded), 3,693 from NHS, and 2,428 from HPFS—were included in the current analysis (Table 1). MDACC melanoma patients tended to have lighter colored skin, eyes, and hair and lower tanning ability than did the MDACC controls (Table 1, all *P* < 0.0001). Similar results were observed in the NHS and HPFS data sets where data were available. Data on family history of melanoma were not collected in the MDACC population; melanoma patients had a higher percentage of family history of melanoma than controls in both the NHS and HPFS data sets (Table 1, all *P* < 0.0001).

**Table 1:** Demographic characteristics of the study participants*.

	**MD Anderson Study, No. (%) (n = 2,829)^&^**			**Harvard Nurse Health Study, No. (%) (n = 3,693)**			**Harvard Health Professionals Follow-Up Study, No. (%) (n = 2,428)**		
**Characteristic**	**Melanoma Patients(1,804)**	**Controls(1,025)**	***P* Value**	**Melanoma Patients(317)**	**Controls(3,376)**	***P* Value**	**Melanoma Patients(177)**	**Controls(2,251)**	***P* Value**
Sex									
Male	1,059 (58.7)	613 (59.8)	0.5989	0 (0)	0 (0)	-	177 (100.0)	2,251 (100.0)	-
Age, y (mean ± SD)	52.1 ± 14.5	51.3 ± 12.6	0.1288	56.8 ± 6.8	57.2 ± 6.7	0.3241	61.2 ± 9.3	61.3 ± 8.5	0.7972
Skin color			< 0.0001			-			-
Light	553 (59.5)	457 (44.5)		-	-		-	-	
Medium	340 (36.6)	490 (47.8)		-	-		-	-	
Dark	37 (4.0)	79 (7.7)		-	-		-	-	
Eye color			< 0.0001			-			0.0947
Blue/gray	409 (44.0)	350 (34.1)		-	-		63 (39.1)	746 (35.8)	
Brown	189 (20.3)	313 (30.5)		-	-		39 (24.2)	676 (32.4)	
Hazel/green	332 (35.7)	363 (35.4)		-	-		59 (36.7)	663 (31.8)	
Hair color			< 0.0001			< 0.0001			0.0169
Blonde	234 (25.9)	193 (18.9)		51 (16.8)	365 (11.4)		26 (16.2)	242 (11.6)	
Red	89 (9.8)	38 (3.7)		23 (7.6)	110 (3.4)		4 (2.5)	55 (2.6)	
Brown	541 (59.8)	732 (71.8)		225 (74.3)	2,655 (82.6)		121 (75.2)	1,589 (76.3)	
Black	41 (4.5)	57 (5.6)		4 (1.3)	83 (2.6)		10 (6.2)	196 (9.4)	
Tanning ability			< 0.0001						-
Practically none	62 (6.7)	120 (11.7)		35 (11.7)	235 (7.4)		-	-	
Light tan	186 (20.2)	311 (30.3)		94 (31.4)	677 (21.3)		-	-	
Average tan	340 (36.8)	322 (31.4)		119 (39.8)	1,470 (46.3)		-	-	
Deep tan	335 (36.3)	273 (26.6)		51 (17.1)	796 (25.1)		-	-	
Family history						< 0.0001			< 0.0001
First-degree relative with melanoma	-			63 (19.9)	265 (7.9)		22 (12.4)	111 (4.9)	

^*^ Data in the table were not available for all patients and controls for pigmentation characteristics and family history in the MDACC and Harvard data sets.

& Pigmentation information was collected in 931 patients and 1026 controls via questionnaire.

### Single SNP Association with Melanoma Risk

We replicated the associations of 11 SNPs with melanoma in the MDACC data set at a nominal significance level of *P*< 0.05 without adjustment for any covariate. 9 SNPs remained associated with melanoma after Bonferroni adjustment for multiple testing of 11 SNPs (Table 2). Only 5 SNPs in the NHS and 2 in the HPFS data sets were associated with melanoma at a nominal significance level of *P*< 0.05. Only one SNP in each validation data set reached significance after adjustment for multiple testing. A meta-analysis of the pooled data sets confirmed association of 5 SNPs with melanoma after correction for multiple testing. 

**Table 2:** Association with melanoma risk for 11 previously identified melanoma risk variants.

**SNP**	**Minor allele frequency**	**Gene**	**Risk allele**	**MD Anderson Study**		**Harvard Nurse Health Study**		**Harvard Health Professionals Follow-Up Study**		Pooled*	
				**OR (95% CI)**	***P* Value**	**OR (95% CI)**	***P* Value**	**OR (95% CI)**	***P* Value**	**OR (95% CI)**	***P* Value**
rs7412746	0.4452	*ARNT*	T	1.18 (1.06-1.31)	2.80 × 10^-3^	1.24 (1.04-1.46)	1.40 × 10^-2^	0.97 (0.78-1.21)	7.79 × 10^-1^	1.15 (1.02-1.29)	1.88 × 10^-2^
rs13016963	0.3834	*ALS2CR12*	A	1.13 (1.01-1.26)	3.56 × 10^-2^	1.04 (0.88-1.23)	6.57 × 10^-1^	0.78 (0.62-0.98)	2.96 × 10^-2^	0.99 (0.81-1.20)	9.13 × 10^-1^
rs4636294	0.4781	*LOC402359*	A	1.23 (1.10-1.37)	2.00 × 10^-4^	1.00 (0.85-1.18)	9.84 × 10^-1^	1.08 (0.87-1.33)	4.98 × 10^-1^	1.11 (0.97-1.28)	1.18 × 10^-1^
rs1335510	0.3919	*LOC100418*	T	1.26 (1.12-1.40)	5.85 × 10^-5^	1.03 (0.87-1.21)	7.66 × 10^-1^	1.03 (0.83-1.28)	8.10 × 10^-1^	1.12 (0.96-1.30)	1.51 × 10^-1^
rs7023329	0.4696	*MTAP*	A	1.26 (1.13-1.41)	2.27 × 10^-5^	1.01 (0.85-1.18)	9.55 × 10^-1^	1.11 (0.90-1.38)	3.41 × 10^-1^	1.13 (0.97-1.32)	1.03 × 10^-1^
rs10830253	0.3398	*TYR*	G	1.19 (1.06-1.33)	3.40 × 10^-3^	1.27 (1.07-1.50)	7.00 × 10^-3^	1.54 (1.24-1.92)	1.00 × 10^-4^	1.29 (1.12-1.48)	2.94 × 10^-4^
rs1801516	0.1333	*ATM*	G	1.23 (1.05-1.44)	1.01 × 10^-2^	1.16 (0.92-1.48)	2.16 × 10^-1^	0.84 (0.62-1.14)	2.57 × 10^-1^	1.10 (0.89-1.34)	3.75 × 10^-1^
rs12913832	0.2250	*HERC2*	G	1.43 (1.26-1.62)	5.60 × 10^-8^	1.23 (1.01-1.49)	3.80 × 10^-2^	1.12 (0.88-1.43)	3.44 × 10^-1^	1.29 (1.12-1.48)	4.58 × 10^-4^
rs258322	0.1203	*CDK10*	A	1.54 (1.29-1.84)	1.41 × 10^-6^	1.62 (1.28-2.05)	6.05 × 10^-5^	1.28 (0.90-1.82)	1.70 × 10^-1^	1.53 (1.34-1.74)	2.51 × 10^-10^
rs4911442	0.1506	*ASIP*	G	1.27 (1.08-1.49)	3.30 × 10^-3^	1.30 (1.03-1.65)	2.86 × 10^-2^	1.32 (0.96-1.83)	8.58 × 10^-2^	1.28 (1.14-1.45)	5.44 × 10^-5^
rs132985	0.4518	*PLA2G6*	C	1.20 (1.08-1.34)	1.20 × 10^-3^	1.12 (0.95-1.32)	1.84 × 10^-1^	1.20 (0.96-1.49)	1.04 × 10^-1^	1.18 (1.08-1.28)	1.45 × 10^-4^

SNP, single-nucleotide polymorphism

^*^ A random effect model was used to pool the 3 data sets in meta-analysis.

### Association between PRS and Melanoma

The distribution of the total number of risk alleles across 11 SNPs was symmetric among the MDACC melanoma patients and matched controls (range from 2 to 19 risk alleles); patients carried more risk alleles than did controls (Figure S1 in File S1). Participants in the MDACC data set carrying 15 or more risk alleles were more than 5 times as likely to have melanoma than were those carrying 6 or fewer risk alleles (OR = 5.12, 95% CI, 3.20-8.21; *P* < 0.0001; Table S3 in File S1). Per-unit increase of PRS was associated with higher risk for melanoma (OR = 1.17, 95% CI, 1.12-1.22; *P* = 2.17 × 10^-13^) after adjustments for other covariates in the MDACC data set, and the result was replicated in the NHS and HPFS data sets. In the pooled data set, per-unit increase of PRS led to a 1.12-time increase in melanoma risk (95% CI, 1.06-1.18; *P* = 4.63 × 10^-5^). Participants in the highest tertile of PRS in the MDACC data set were 2.13 times more likely to have melanoma than those in the lowest tertile of PRS (95% CI, 1.69-2.68; *P* = 1.41 × 10^-10^); this result was confirmed only in the NHS data set (OR = 1.55, 95% CI, 1.14-2.11; *P* = 5.00 × 10^-3^). A meta-analysis of the three data sets confirmed an association of PRS with melanoma susceptibility (OR = 1.69, 95% CI, 1.28-2.25; *P* = 2.24 × 10^-4^; Table 3). To observe whether participants with extreme values of PRS had a high risk of having melanoma, we divided the participants into 2 groups at a threshold of 95% of PRS. After adjustments for sex, age, and pigmentation, we found that those with a PRS above 95% had a 2.34-times increased risk of having melanoma compared with those with a PRS below 95% (95% CI, 1.40-3.89; *P*=0.0011). In summary, PRS as a continuous variable identified individuals with a relatively high risk for melanoma susceptibility. No individual SNP in Table 2 has an OR as high as 2.34. Therefore, joint analysis of multiple variants identified individuals with a very high risk for melanoma, which was not accomplished through any single SNP test.

**Table 3:** Association between weighted polygenic risk score (PRS) and melanoma risk in 3 data sets.

	Univariate analysis			Multivariate analysis		
Study	No.	OR for PRS (95% CI)	*P* Value	No.	OR for PRS (95% CI)	*P* Value
Continuous values						
MDACC^*^	2,830	1.19 (1.15-1.24)	3.71 × 10^-23^	1,949	1.17 (1.12-1.22)	2.17 × 10^-13^
NHS^†^	3,693	1.13 (1.08-1.19)	1.42 × 10^-6^	3,462	1.10 (1.04-1.16)	4.00 × 10^-4^
HPFS^‡^	2,428	1.10 (1.03-1.18)	4.80 × 10^-3^	2,237	1.07 (1.00-1.15)	6.91 × 10^-2^
Pooled^§^	8,951	1.15 (1.09-1.20)	5.39 × 10^-8^	7,468	1.12 (1.06-1.18)	4.63 × 10^-5^
Tertile 3 vs. Tertile 1						
MDACC^*^	2,830	2.34 (1.93-2.84)	5.46 × 10^-18^	1,949	2.13 (1.69-2.68)	1.41 × 10^-10^
NHS^†^	3,693	1.73 (1.30-2.30)	2.00 × 10^-4^	3,462	1.55 (1.14-2.11)	5.00 × 10^-3^
HPFS^‡^	2,428	1.54 (1.06-2.24)	2.51 × 10^-2^	2,237	1.34 (0.90-2.00)	1.57 × 10^-1^
Pooled^¶^	8,951	1.90 (1.46-2.48)	1.92 × 10^-6^	7,468	1.69 (1.28-2.25)	2.24 × 10^-4^

^*^ Adjusted for age, sex, skin color (light, medium, dark), eye color (blue/gray, brown, hazel/green), hair color (blonde, red, brown, black), and tanning ability (always, usually, moderate, minimal, rarely/never) in the multivariate analysis.

^†^ Adjusted for age, hair color (blonde, red, brown, black), tanning ability (practically none, light tan, average tan, deep tan), and family history in the multivariate analysis.

^‡^ Adjusted for age, hair color (blonde, red, brown, black), eye color (blue/gray, brown, hazel/green), and family history in the multivariate analysis.

^§^ *P* value for Cochrane Q statistic equals 0.0536, I^2^ heterogeneity index equals 65.83.

^¶^ *P* value for Cochrane Q statistic equals 0.0807, I^2^ heterogeneity index equals 60.28.

We additionally investigated whether the association between PRS and melanoma was driven by the most significant SNP in the MDACC data set (rs12913832 in *HERC2*). After removing rs12913832 in a multivariate analysis of the MDACC data set, the OR for the top tertile of PRS decreased only slightly, from 2.13 to 2.11. Therefore, the observed association between PRS and melanoma was not uniquely accounted for by this individual SNP. 

Because most common genetic variants for melanoma susceptibility are also associated with phenotypic risk factors such as pigmentation or nevi, we further observed the relationship between PRS and each pigmentation factor. We can find that PRS only accounted for 1-3.6% of variation of pigmentation (all P-values<0.0001, Table S4 in File S1), indicating that PRS was not fully correlated with skin color, eye color or hair color but conferred effect on both pigmentation factors and melanoma risk. Hence in the multivariate analysis, we observed the effect of PRS on melanoma susceptibility with adjustment for conventional phenotypic factors including pigmentation factors and expected to determine if the genetic variants can contribute additionally to risk prediction beyond conventional phenotypic factors. 

**Figure 1:**
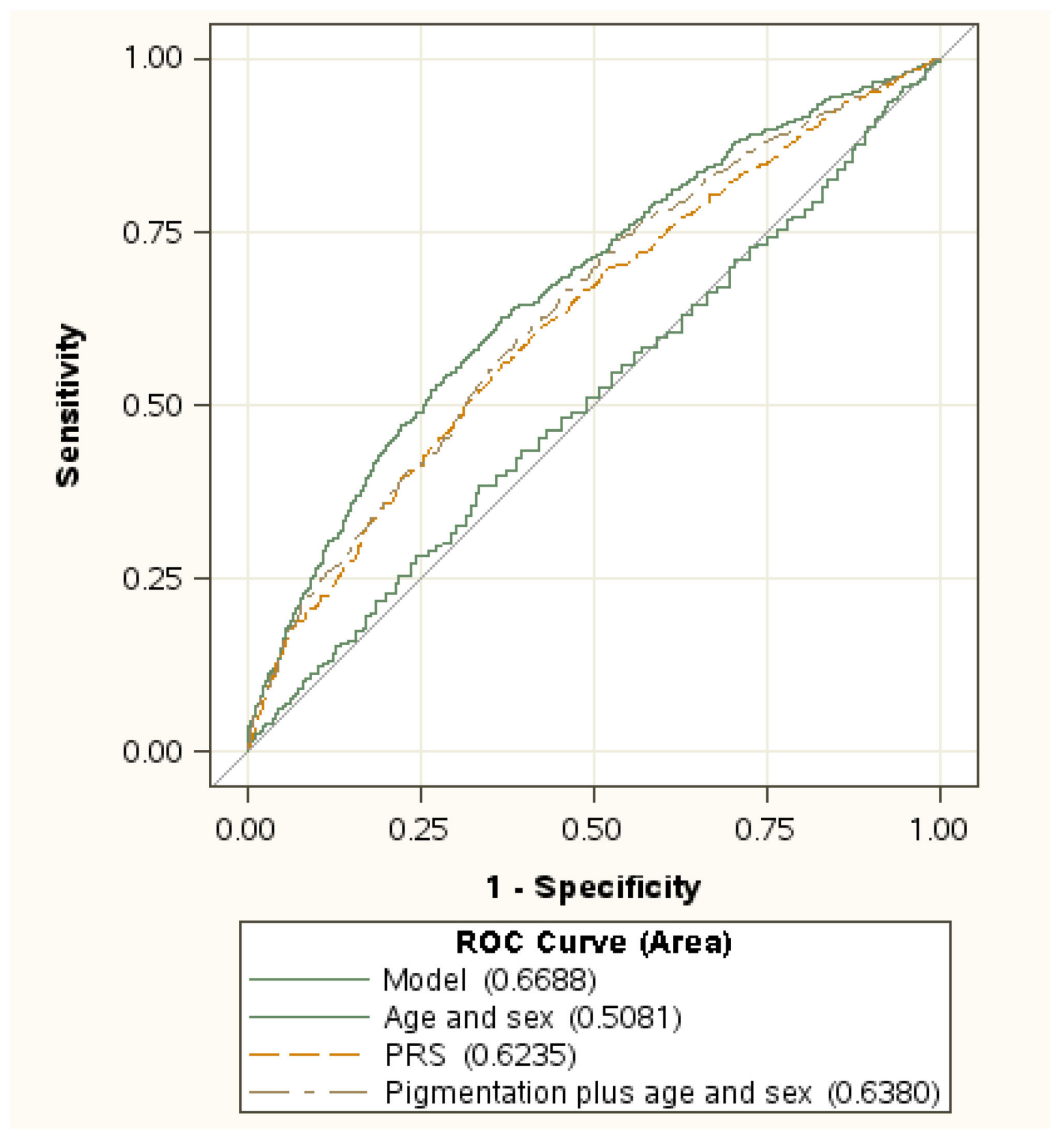
Receiver operating characteristic (ROC) curve for prediction of melanoma risk based on age and sex; pigmentation, age, and sex; polygenic risk score (PRS); and PRS plus pigmentation, age, and sex(model).

The performance of our PRS model incorporating the measured effect of 11 SNPs was compared with the most significant SNP rs12913832 or other risk models using the MDACC data set (Figure 1 and Table 4). The overall discrimination of the PRS for melanoma was 0.62 (95% CI, 0.60-0.65), which was higher than that for the model with rs12913832 (AUC=0.55, 95% CI, 0.53-0.57) and then that for the model consisting of sex and age (AUC=0.51, 95% CI, 0.48-0.53), but approximately the same as that for the standard phenotypic model consisting of sex, age, and pigmentation (AUC=0.64, 95% CI, 0.61-0.66). Adding PRS to the conventional risk model offered a small but significant improvement to melanoma risk prediction (increase of AUC = 0.03, 95% CI, 0.02-0.05; *P* = 6.01 × 10^-5^). The overall discrimination of the PRS plus conventional phenotypic factors was 0.69 (95% CI, 0.64-0.69) in the MDACC data set. The overall discrimination of the PRS for melanoma was higher in the MDACC data set than that in the NHS and HPFS data sets, probably due to the larger sample size of melanoma patients in the former data set (Table S5 in File S1).

**Table 4:** Risk prediction performance for different sets of predictors in the MD Anderson data set.

**Model**	**AUC (95% CI)**	**Model Contrast**	**ROC Contrast Estimation**	***P* Value**
① rs12913832	0.55(0.53-0.57)	-	-	-
②PRS	0.62 (0.60-0.65)	②-①	0.07(0.05-0.07)	1.53× 10^-10^
③Sex + age	0.51 (0.48-0.53)	-	-	-
④Sex + age + pigmentation	0.64 (0.61-0.66)	④-③	0.13 (0.10-0.16)	1.83 × 10^-11^
⑤Sex + age + pigmentation + PRS	0.69 (0.64-0.69)	⑤-④	0.03 (0.02-0.05)	6.01 × 10^-5^

AUC, area under the receiver operating characteristic curve; PRS, polygenic risk score; ROC, receiver operating characteristic.

Results for reclassification of melanoma risk using the PRS score in the MDACC data set are shown in Table 5. One hundred thirteen control participants (11.0%) classified as having a 50% or greater melanoma risk by the conventional model were reclassified as having a 20% to 50% risk when PRS was added to the model; 306 melanoma patients (17.0%) classified as having a 20% to 50% risk were reclassified as having a 50% or greater risk when PRS was incorporated. In addition, 231 controls predicted to be at moderate risk (20%-50%) for melanoma according to the model without genotypic data were reclassified as high risk (>50%) in the model including genotype. NRI was not significant (0.0109; *P* = 0.6076), but IDI, which estimates the new model’s improvement in average sensitivity without sacrificing average specificity, was significant (0.0350; *P* < 0.0001), indicating that the addition of PRS on average might improve the predictive ability of a clinical risk model using conventional risk factors.

**Table 5:** Reclassification of participants’ predicted melanoma risk with and without weighted genetic variants in MD Anderson Study ^*^.

**Predicted Risk** ^†^	**Predicted Risk Based on Known Risk Factors and PRS**			
Controls				
	**<20%**	**20-50%**	**≥ 50%**	
<20%	0	0	0	
20-50%	4 (0.4)	579 (56.4)	231 (22.5)	
≥ 50%	0	113 (11.0)	99 (9.7)	
Patients				
<20%	0	0	0	
20-50%	0	372 (20.6)	306 (17.0)	
≥ 50%	0	86 (4.8)	1,039 (57.6)	
**Net Reclassification Improvement of PRS for Melanoma Risk**	**Up**	**Down**	**NRI**	***P* Value**
Patients	306	86	0.1220	<0.0001
Controls	231	117	0.1111	<0.0001
Total	-	-	0.0109	0.6076

PRS, polygenic risk score; NRI, net reclassification improvement.

* Integrated discriminant index equal to 0.0350 (*P* < 0.0001).

† Predicted risk based on age, sex, eye color, skin color, hair color, and tanning ability.

## Discussion

In a polygenic risk model, the joint effect of 11 independent SNPs previously identified by GWAS significantly predicted melanoma risk independent of traditional demographic and pigmentation factors. The PRS improved the discrimination of melanoma risk by a small margin compared to risk assessment based on standard phenotypic risk factors. Our results suggest that a risk model built with a modest set of biologic objectively and directly measured risk factors, including a PRS, could ultimately be incorporated into an improved model of melanoma risk to supplement or even replace conventional phenotypic risk factors, including assisting in risk stratification to improve the efficacy and cost-effectiveness of large-scale population-based screening programs. It is acknowledged, however, that PRS in this study provided only a small additional benefit in prediction and discrimination over conventional phenotypic assessment; PRS was confirmed to be in large part a surrogate measure of standard phenotypic parameters for melanoma risk prediction because most common variants in our study confer effects on pigmentation or nevi as well as melanoma risk. 

The National Cancer Institute developed a melanoma risk prediction model by assessing demographics, pigmentation and sun exposure factors among 718 white patients and 945 matched controls in Philadelphia and San Francisco[26]. Using the constructed model, the AUC was 0.70 for women 50 years or older and 0.80 for men 20 to 49 years old respectively. European investigators constructed a model that included common nevi, skin and hair color, freckles, and sunburns in childhood, and in an external validation of the model’s performance, the AUC was 0.79[27]. The prediction model we built using MDACC phenotype data yielded an AUC of 0.64, lower than previous studies. The reason for the lower AUC in MDACC risk model might be that the current analysis didn’t include the total number of nevi and presence of dysplastic nevi, and/or because of differences in the population structure of the cohorts investigated. The performance of the conventional risk model included in the current study was improved by including PRS, although the magnitude of improvement was small. Our risk model using only PRS had discriminability approximately equal to that of a conventional phenotypic model. We acknowledge that the current relatively high costs associated with genotyping and the relative low cost and convenience associated with phenotypic assessment of individual patients argues against routine incorporation of genotyping into melanoma risk assessment of patients seen in clinical practice. However, direct genotyping is increasingly straightforward to perform at rapidly decreasing cost; furthermore, accurate phenotypic assessment of large numbers of patients in population-based screening programs may be associated with significant costs of its own. In such a scenario, especially if additional biologic measures of risk can also be incorporated a model that includes limited genotyping to provide a PRS with discrimination at least equal to that obtained from interview data from questionnaires or from direct physical examination could result in risk prediction at least as accurate as a standard phenotypic model and at potentially similar or even reduced cost.

We built our model to minimize effects of linkage disequilibrium, and we assumed no or weak linkage disequilibrium between different variants in the model. We ran stepwise selection on our discovery data set and found that only 9 SNPs still remained in the final logistic regression model at significance level of *P*<0.05; the performance of risk prediction based on those 9 SNPs was close to that based on all 11 SNPs. To save genotyping cost, one could potentially reduce the PRS to include only optimal SNPs instead of the complete set for genetic testing. Our PRS risk model with 11 SNPs did not include any MC1R variants because they were not available in the validation datasets. We included the adjacent CDK10 SNP rs258322, which is in linkage disequilibrium with the MC1R gene on chromosome 16. In the MDACC data set, the most significant MC1R SNP (rs1805007) accounted for the entire effect of the most significant CDK10 SNP (rs258322) (data not shown). Therefore, we predict that an alternative model that included this MC1R SNP would perform approximately as well as the model including the CDK10 SNP. To test whether the joint effect of multiple variants was dominated by the most significant SNP, we adjusted for the most significant SNP in the MDACC data set (rs12913832) and found that the combined effect of the remaining variants remained significant, with only a small decrease in the OR; thus, the predictive ability of PRS is not solely due to the effect of the most significant SNP. 

Our study has limitations and therefore should be considered preliminary, even with external validation. First, both the discovery and validation groups were drawn from non-Hispanic white populations in the United States; the model constructed here in USA communities may not be applicable to other ethnic groups in other countries. Second, the relatively small number of cases from the Harvard Nurse Health study and the follow-up study limits the power of our validation analyses. In addition, the absence of males in the Harvard Nurse Health study and the absence of females in the Follow-Up study could lead to gender bias. For this reason, when we combined the three datasets, we first adjusted for gender in the MDACC dataset and then pooled the results using a random-effect model in meta-analysis; this approach can minimize gender bias. Third, our approach focused on common variants with weak effect but did not incorporate much rarer variants with higher effect. Fourth, our analysis did not take into account gene-gene and gene-environmental interactions that widely exist in complex diseases such as cancer[28]. Fourth, our risk prediction model was only built in non-Hispanic whites. We assumed there was no population substructure, and ethnicity was not adjusted for in our analysis. Finally, there are ethical and legal issues associated with genetic testing and risk prediction that would need to be addressed before polygenic risk prediction could be implemented on a population-wide basis[29]. 

 In summary, these results while preliminary demonstrate that joint analysis of multiple common, generally low-penetrance SNPs can identify individuals with a very high risk for melanoma susceptibility, and suggest that a polygenic profile could ultimately be incorporated into an improved tool for melanoma risk assessment that could supplement or eventually even replace standard phenotypic variables, including for population-based screening. Future investigations should include polygenic profiling together with standardized phenotypic assessment in prospective trials of melanoma screening and prevention, and investigate the effects of the addition of other genetic, epigenetic and biologic markers to standard melanoma risk models.

## Supporting Information

File S1 **Combined Supporting Information containing a description of the previously published SNPs for melanoma risk, their associations with melanoma susceptibility in MDACC study, correlation between polygenetic risk score (PRS) and pigmentation, and risk prediction using PRS.**
(PDF)
