# Supplementary material for: Joint Effect of Multiple Common SNPs Predicts Melanoma Susceptibility
Source: PLoS One. 2013 Dec 31;8(12):e85642. doi: 10.1371/journal.pone.0085642 (PMC3877376; doi:10.1371/journal.pone.0085642)
Supplement: File S1 — Combined Supporting Information containing a description of the previously published SNPs for melanoma risk, their associations with melanoma susceptibility in MDACC study, correlation between polygenetic risk score (PRS) and pigmentation, and risk prediction using PRS. (PDF) [file pone.0085642.s001.pdf]

Table S1. 38 previously reported significant SNPs and their association in MDACC data.

SNP	MAF	chromosome	position	riskalle	Freq	AlleleOddsRatio	ProbAllele	GeneSymbol	Location
rs7412746	0.4452	1	149127095	T	0.5548	0.844722	0.00238943	ARNT	flanking_5UTR
rs13016963	0.3834	2	201871056	A	0.3834	1.127217	0.03614075	ALS2CR12	
rs16891982	0.0222	5	33987450	G	0.9778	0.572441	0.00325339	SLC45A2	coding
rs35391	0.01524	5	33991430	G	0.98476	0.579479	0.01619985	SLC45A2	intron
rs2877	0.3031	7	82602361	C	0.3031	1.040979	0.50454592	PCLO	
rs1408799	0.302	9	12662097	C	0.698	1.024851	0.68322462	TYRP1	flanking_5UTR
rs4636294	0.4781	9	21737803	A	0.5219	1.228683	0.00019826	LOC402359	intron
rs2218220	0.4774	9	21746089	C	0.5226	1.230055	0.00018304	LOC100418	intergenic
rs1335510	0.3919	9	21747803	T	0.6081	0.796941	5.5934E-05	LOC100418	intergenic
rs935053	0.4732	9	21773922	G	0.5268	0.799481	5.271E-05	MTAP	
rs10757257	0.383	9	21796564	G	0.617	0.790754	3.2524E-05	MTAP	intron
rs7023329	0.4696	9	21806528	A	0.5304	1.268512	1.742E-05	MTAP	intron
rs1485993	0.3672	11	69071595	A	0.3672	1.097952	0.10467176	CCND1	
rs1042602	0.3673	11	88551344	C	0.6327	0.898588	0.06256211	TYR	coding
rs1393350	0.3074	11	88650694	A	0.3074	1.162303	0.01280764	TYR	intron
rs10830253	0.3398	11	88667691	G	0.3398	1.189011	0.00322936	TYR	
rs1806319	0.3873	11	88677584	C	0.3873	1.16962	0.00597628	TYR	flanking_3UTR
rs1801516	0.1333	11	107680672	G	0.8667	0.816051	0.01047072	ATM	coding
rs12896399	0.4637	14	91843416	T	0.4637	0.944094	0.29992929	SLC24A4	flanking_5UTR
rs1800407	0.07881	15	25903913	G	0.92119	0.95082	0.61973629	OCA2	coding
rs1129038	0.2263	15	26030454	A	0.7737	1.434767	2.7017E-08	HERC2	3UTR
rs12913832	0.225	15	26039213	G	0.775	0.699819	4.0496E-08	HERC2	intron
rs258322	0.1203	16	88283404	A	0.1203	1.556391	8.6465E-07	CDK10	intron
rs1805006	0.0121	16	88513419	A	0.0121	1.648471	0.06623304	MC1R	coding
rs1805007	0.1329	16	88513618	T	0.1329	0.57257	2.9377E-09	MC1R	coding
rs1805008	0.09404	16	88513645	A	0.09404	1.392038	0.00076005	MC1R	coding
rs17305657	0.102	20	31270249	C	0.102	1.191345	0.06160779	C20orf71	intron
rs4911414	0.3674	20	32193105	T	0.3674	0.904762	0.08232156	LOC729547	flanking_3UTR
rs1015362	0.2691	20	32202273	C	0.7309	1.068199	0.28675368	RPS2P1	intergenic
rs910873	0.1128	20	32635433	A	0.1128	1.404199	0.00021695	PIGU	intron

rs17305573	0.1128	20	32643813	C	0.1128	1.404199	0.00021695	PIGU	intron
rs4911442	0.1506	20	32818707	G	0.1506	0.792153	0.00353487	ASIP	intron
rs1885120	0.1081	20	33040650	C	0.1081	1.413233	0.00021614	MYH7B	intron
rs45430	0.3765	21	41667951	T	0.6235	0.905962	0.08258403	MX2	intron
rs2284063	0.3521	22	36874244	A	0.6479	1.17892	0.00436978	PLA2G6	intron
rs6001027	0.3424	22	36875565	A	0.6576	1.181292	0.00404126	PLA2G6	intron
rs132985	0.4518	22	36893417	C	0.5482	1.194021	0.00139323	PLA2G6	intron
rs11547464	-	16	-	-	-	1.670447	0.10781411		

Table S2. Risk allele effect from previous studies (using risk allele to count and their beta coefficients as weights).

SNP	MAF	Chromosome	Position	Risk Allele	Frequency	Allele OR (95% CI)	Beta OR	P-value for risk allele in the reported study	Gene Symbol	Reference	Sample
**rs7412746**	**0.4452**	**1**	149127095	**T**	**0.5548**	**1.15**	**0.1393**	**9.0×10^{-10}**	***ARTN***	**(MacGregor *et al.*, 2011)**	**Discovery: 2,168 patients + 4,387 controls; Validation: 2,804 + 1,804 - 585 patients + 7,618 + 1,026 + 6,500 controls**
**rs13016963**	**0.3834**	**2**	201871056	**A**	**0.3834**	**1.14 (1.09-1.19)**	**0.13103**	**8.6×10^{-10}**	***CASP8***	**(Barrett *et al.*, 2011)**	**Discovery: 2,981 patients + 1,982 specific controls + 6,426 additional controls; 2,804 patients + 1,835 + 5,783 controls**
**rs4636294**	**0.4781**	**9**	**21737803**	**A**	**0.5219**	**1.16 (1.09-1.23)**	**0.14842**	**1.97×10^{-6}**	**LOC402359**	**(Bishop *et al.*, 2009)**	**Discovery: 1,650 patients + 4,336 controls; Validation 1: 1,149 patients + 964 controls; Validation 2: 1,163 patients + 903 controls**
rs4636294	0.4781	9	21737803	A	0.5219	1.21 (1.14-1.28)		3.7×10^{-8}	LOC402359	(Falchi *et al.*, 2009)	3,131 patients and 2,576 controls
rs1335510	0.3919	9	21747803	T	0.6081	1.19 (1.11-1.30)		1.14×10^{-4}	LOC100418	(Bishop *et al.*, 2009)	Discovery: 1,650 patients + 4,336 controls
**rs1335510**	**0.3919**	**9**	**21747803**	**T**	**0.6081**	**1.19 (1.12-1.25)**	**0.17395**	**1.1×10^{-7}**	**LOC100418**	**(Falchi *et al.*, 2009)**	**3,131 patients + 2,576 controls**
**rs7023329**	**0.4696**	**9**	**21806528**	**A**	**0.5304**	**1.18(1.10-1.25)**	**0.16551**	**4.03×10^{-7}**	***MTAP***	**(Bishop *et al.*, 2009)**	**Discovery: 1,650 patients + 4,336 controls; Validation 1: 1,149 patients + 964 controls; Validation 2: 1163**

											**patients + 903 controls**
rs7023329	0.4696	9	21806528	A	0.5304	1.20 (1.13-1.26)		2.7×10^{-7}	*MTAP*	(Falchi *et al.*, 2009)	3131 patients + 2,576 controls
**rs10830253**	**0.3398**	**11**	**88667691**	**G**	**0.3398**	**1.26 (1.14-1.39)**	**0.23111**	$\mathbf{2.81 \times 10^{-6}}$	***TYR***	**(Bishop *et al.*, 2009)**	**Validation 1: 1,149 patients + 964 controls; Validation 2: 1,163 patients + 903 controls**
**rs1801516**	**0.1333**	**11**	107680672	**G**	**0.8667**	**1.19 (1.11-1.28)**	**0.17395**	$\mathbf{3.4 \times 10^{-9}}$	***ATM***	**(Barrett *et al.*, 2011)**	**Discovery: 2,981 patients + 1,982 specific controls + 6,426 additional controls; 2,804 patients + 1,835 + 5,783 controls**
**rs12913832**	**0.225**	**15**	**26039213**	**G**	**0.775**	**1.15**	**0.13976**	**0.042**	***HERC2***	**(Duffy *et al.*, 2010)**	**1,483 casespatients and+ 3,098 controls**
**rs258322**	**0.1203**	**16**	**88283404**	**A**	**0.1203**	**1.6 7(1.52-1.83)**	**0.51282**	$\mathbf{2.54 \times 10^{-27}}$	***CDK10***	**(Bishop *et al.*, 2009)**	**Discovery: 1,650 patients + 4,336 controls; Validation 1: 1,149 patients + 964 controls Validation 2: 1,163 patients + 903 controls**
rs4911442	0.1506	20	32818707	G	0.1506	1.48 (1.24-1.77)		1.80×10^{-5}	*ASIP*	(Bishop *et al.*, 2009)	Validation 1: 1,149 patients + 964 controls; Validation 2: 1,163 patients + 903 controls
**rs4911442**	**0.1506**	**20**	**32818707**	**G**	**0.1506**	**1.51 (1.33-1.70)**	**0.41211**	$\mathbf{2.86 \times 10^{-11}}$	***ASIP***	**(Brown *et al.*, 2008)**	**2,019 patients + 2105 controls**
**rs132985**	**0.4518**	**22**	**36893417**	**C**	**0.5482**	**1.23 (1.15-1.30)**	**0.20701**	$\mathbf{2.6 \times 10^{-7}}$	***PLA2G6***	**(Falchi *et al.*, 2009)**	**3,131 patients + 2,576 controls**

MAF, minor allele frequency; SNP, single-nucleotide polymorphism

Bold rows indicate the study with the largest sample size if an allele was identified in multiple studies

Table S3. Odds ratios for melanoma risk according to the number of risk alleles carried in the MDACC data set.

**Number of risk alleles**	**Frequency(percentage)**	**Odds Ratio**	**95% confidence interval**
0-6	112(5.74)	1.00	-
7	113(5.79)	1.70	1.08-2.70
8	168(8.62)	1.62	1.06-2.45
9	238(12.21)	1.73	1.16-2.58
10	284(14.56)	2.27	1.54-3.35
11	263(13.49)	2.29	1.55-3.38
12	269(13.79)	3.17	2.14-4.70
13	218(11.18)	2.56	1.70-3.85
14	149(7.64)	5.02	3.15-8.00
15-	136(6.97)	5.12	3.20-8.21

Table S4. Correlation between polygenetic risk score and pigmentation factors*

**Model**	**MDACC study**		**NHS study**		**HPFS**	
	R^2	P-value	R^2	P-value	R^2	P-value
Skin color	0.0259	<0.0001	-	-	-	-
Eye color	0.0340	<0.0001	-	-	0.0361	<0.0001
Hair color	0.0100	<0.0001	0.0244	<0.0001	0.0232	<0.0001

*skin color (light=1-3, medium=4-6, dark=7-10), eye color (blue/gray, brown, hazel/green), hair color (blonde, red, brown, black)

Table S5. Risk prediction performance for different sets of predictors in three studies(AUC (95% CI)).

Model	MDACC study	NHS study	HPFS
1) PRS	0.62 (0.60-0.65)	0.58(0.54-0.61)	0.56(0.51-0.60)
4) pigmentation + PRS + age +sex/family history	0.69 (0.64-0.69)	0.67(0.63-0.70)	0.62(0.58-0.67)

AUC, area under the receiver operating characteristic curve; PRS, polygenic risk score.

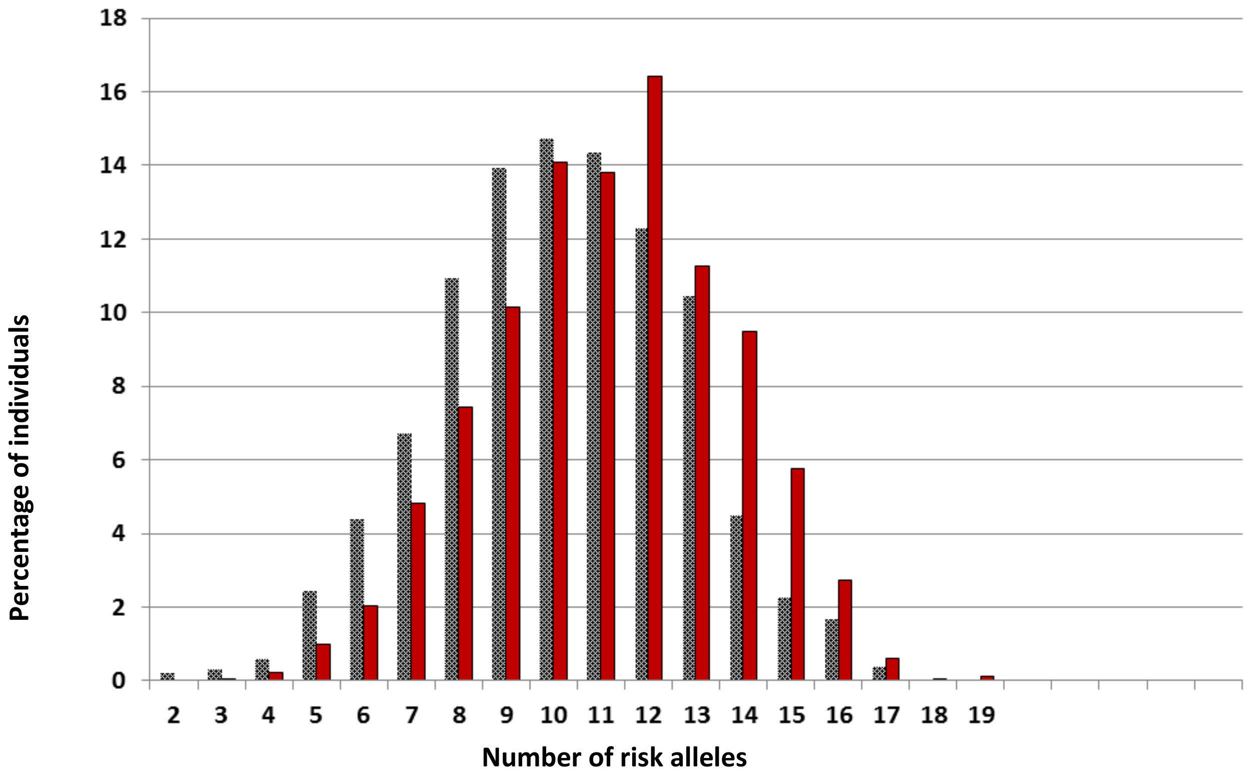

Figure S1. Distribution of risk alleles in melanoma patients (solid bars) and controls (texture bars)

File S1. Combined Supporting Information containing a description of the previously published SNPs for melanoma risk, their associations with melanoma susceptibility in MDACC study, correlation between polygenetic risk score(PRS) and pigmentation, and risk prediction using PRS.
